# The Hematology of Tomorrow Is Here—Preclinical Models Are Not: Cell Therapy for Hematological Malignancies

**DOI:** 10.3390/cancers14030580

**Published:** 2022-01-24

**Authors:** Lorena Arranz

**Affiliations:** 1 Stem Cells, Ageing and Cancer Research Group, Department of Medical Biology, Faculty of Health Sciences, UiT—The Arctic University of Norway, MH2 Building Level 10, 9019 Tromsø, Norway; lorena.arranz@uit.no; 2 Centre for Molecular Medicine Norway (NCMM), Faculty of Medicine, University of Oslo (UiO), 0349 Oslo, Norway

**Keywords:** cellular immunotherapy, cell therapy, adoptive cell therapy, mesenchymal stromal cells, hematopoietic stem cells, hematopoietic malignancy, acute myeloid leukemia, preclinical models

## Abstract

**Simple Summary:**

Cell therapy is revolutionizing the prospect of deadly hematological malignancies such as high-risk acute myeloid leukemia. Stem cell therapy of allogeneic source from compatible human leukocyte antigen donor has exceptional success promoting durable remissions, but the rate of relapse is currently still high and there is transplant-related mortality. This review presents the current knowledge on the clinical use of mesenchymal stromal cells to improve outcomes in hematopoietic stem cell transplants. As an alternative or adjuvant approach to prevent relapse, we summarize the status of the promising forms of cellular immunotherapy aimed at targeting not only the bulk but also the cells of origin of leukemia. Finally, we discuss the available in vivo models for disease modelling and treatment efficacy prediction in these contexts.

**Abstract:**

The purpose of this review is to present the current knowledge on the clinical use of several forms of cell therapy in hematological malignancies and the preclinical models available for their study. In the context of allogeneic hematopoietic stem cell transplants, mesenchymal stromal cells are pursued to help stem cell engraftment and expansion, and control graft versus host disease. We further summarize the status of promising forms of cellular immunotherapy including CAR T cell and CAR NK cell therapy aimed at eradicating the cells of origin of leukemia, i.e., leukemia stem cells. Updates on other forms of cellular immunotherapy, such as NK cells, CIK cells and CAR CIK cells, show encouraging results in AML. The considerations in available in vivo models for disease modelling and treatment efficacy prediction are discussed, with a particular focus on their strengths and weaknesses for the study of healthy and diseased hematopoietic stem cell reconstitution, graft versus host disease and immunotherapy. Despite current limitations, cell therapy is a rapidly evolving field that holds the promise of improved cure rates, soon. As a result, we may be witnessing the birth of the hematology of tomorrow. To further support its development, improved preclinical models including humanized microenvironments in mice are urgently needed.

## 1. Introduction: Cell Therapy in a Nutshell

Cell therapy is a treatment grounded on delivery of viable cells that are injected or transplanted into the patient to exert a therapeutic effect. There are two main principles for the therapeutic action; either replacement of diseased cells with healthy, functional ones, or capacity to exert or induce cell-mediated immunity and/or release soluble factors such as cytokines, chemokines and growth factors that function in paracrine/endocrine ways on diseased cells.

### 1.1. Hematopoietic Stem Cells

Examples of cell replacement therapies include blood transfusions, organ transplants and bone marrow (BM) transplants, but a prospect of cure needs to always meet the requirement of replacing the dysfunctional stem cells, i.e., stem cell therapy. Unfortunately, adult stem cell treatments have often been propelled by empty promises [1], with few exceptions such as the BM transplant. This is an exceptionally successful stem cell therapy applied frequently in the clinic for a variety of severe diseases. The stem cell therapy of allogeneic source from compatible human leukocyte antigen (HLA) donor has improved dramatically the rates of durable remission in patients of severe hematological diseases such as high-risk acute myeloid leukemia (AML) [2], but the rate of relapse is still high and the primary cause of death [3,4], and there is a need to reduce the transplant-related mortality [5]. Reduced-intensity conditioning regimens have been tested to reduce the non-disease mortality and open this alternative for the older patient [6,7,8], but they seem less effective in disease control, particularly for higher-risk AML [9,10,11]. Monoclonal antibodies, including combination of anti-c-Kit antibody that depletes HSC with blockade of CD47, a myeloid-specific immune checkpoint which extends HSC clearance, show promising results in preclinical studies in mice and non-human primates [12,13]. This combination has been taken into a Phase Ib clinical trial by Forty Seven, Inc. (Menlo Park, CA, USA). A recent follow-up to this strategy combined pharmacologic Janus kinase 1/2 inhibition with anti-CD45 or anti-c-Kit antibodies to enable robust multilineage alloengraftment in mice, balancing graft-versus-host disease (GvHD) and graft-versus-leukemia (GvL) responses [14]. These kinds of approaches promise a future of stem cell transplants without chemotherapy or irradiation, and come along with other new cell therapy strategies that are pursued to reduce relapse and unrelated mortality due to transplant toxicity for patients, as follows.

### 1.2. Mesenchymal Stromal Cells

**Figure 1:**
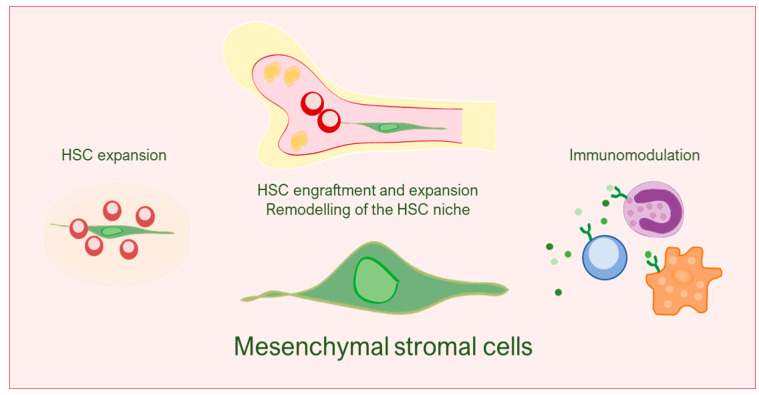
Mesenchymal stromal cells as cell therapy in hematology. Mesenchymal stromal cells are pursued to promote HSC function, remodel the HSC niche in the bone marrow and modulate inflammation.

In hematopoietic stem cell (HSC) transplantation, preconditioning via irradiation or chemotherapeutics is performed to deplete the host diseased stem cells and allow donor HSC engraftment. Without preconditioning, donor chimerism is not established or very low [15]. However, preconditioning of this kind also disrupts niche function by damaging several components, including mesenchymal stromal cells (MSC) [16,17] and results in radiation-induced bystander effects that impair long-term HSC reconstitution by oxidative DNA damage [18]. The HSC niche is a dynamic entity that supports the function, fate and numbers of HSC in the BM, where HSC reside [19]. Mesenchymal stromal cells (MSC) are essential components of the HSC niche, yet the absence of selective markers to study them in vivo has challenged our understanding of their nature [20,21]. Due to their role in tissue remodeling and cell regulation of other cell subsets, particularly stem cells but also immune cells, through dynamic and paracrine interactions, MSC are extensively pursued as therapeutics [21,22,23]. In the context of hematology, MSC are pursued mainly to help promote BM HSC niche reconstitution and HSC engraftment and expansion, treat autoimmune diseases and control GvHD (Figure 1) [24,25]. Preclinical models show a certain degree of success [25], but the malpractice of MSC therapies in the clinic, many times used lacking confirmation on safety and effectiveness from large-scale clinical trials, threatens development of MSC therapeutics [24,26,27]. In addition, failures have been reported in many early- and late-stage clinical trials [28]. Factors contributing to the failure of MSC in clinical applications may include poor-quality controls and inconsistent characteristics of MSC regarding immune-compatibility, functional heterogeneity and differentiation status, among others [21,24].

### 1.3. Cellular Immunotherapy

Adoptive cell therapy or cellular immunotherapy is a form of cell therapy that uses the cells of the immune system, such as T cells or natural killer (NK) cells, to eliminate cancer cells. Some approaches involve isolation, activation and expansion of immune cells to infuse them back into the patient, such as tumor-infiltrating lymphocyte therapy or natural NK cell therapy. More advanced strategies involve autologous or allogeneic immune cells and additional gene therapy to enhance their function, i.e., engineered T cell receptor therapy, chimeric antigen receptor (CAR) T cell therapy and CAR NK cell therapy. These strategies are revolutionizing the field of hematology and beyond. As of today, there are more than 800 clinical trials using CAR T cells in hematology and oncology. In the era of advanced therapy medicinal products, the fourth generation of CAR T cells combine the direct cancer cell killing of the CAR T cell with the additionally engineered release of transgenic cytokines upon CAR signaling in the targeted tumor tissue. This concept is currently explored with single or combined interleukins (IL) such as IL-7, IL-12, IL-15, IL-18 and IL-23 in early phase clinical trials [29]. The next step of fifth generation CAR, with the additional engineered expression of cytokine receptors such as IL-2 that respond to cytokine signals in the tumor microenvironment, are on their way. This strategy elicits antigen-dependent activation of the JAK-STAT pathway resulting in improved cell proliferation and persistence [30,31]. However, adverse events such as cytokine-release syndrome, neurotoxicity and high rate of relapse still limit broad application [32]. Therefore, most clinical efforts are currently pursued with autologous CAR T cells. Allogeneic CAR T cells have high risk of GvHD, and gene editing through CRISPR system seems to be an efficient strategy to manufacture universal CAR T cells deficient in CD3 and HLA-class [33]. However, the elimination of HLA-class I from CAR T cells could induce the attack from host NK cells, which should be taken into consideration in the design of future therapies [34,35].

NK cells are innate immune cells, tightly regulated by a variety of surface inhibitory receptors, i.e., CD94/natural killer group 2A (NKG2A), inhibitory killer immunoglobulin-like receptors (KIRs) and leukocyte immunoglobulin-like receptors (LIRs), and activating receptors i.e., CD94/NKG2C, NKG2D, activating KIRs and natural cytotoxicity receptors (NCRs) [36,37]. One of their specializations is the recognition of the absence of HLA-proteins, which are often downregulated in malignant or virally infected cells helping escape immune surveillance [38]. Most commonly, NK cells are obtained from donor leukapheresis followed by magnetic cell sorting that depletes for CD3^+^ cells and enriches or not for CD56. Donor CD34^+^ progenitor cells may also be used for ex vivo differentiation into NK cells, including cord blood CD34^+^ cells [37]. CAR NK cells provide an advantageous dual killing-capacity by CAR-dependent and -independent mechanisms, such as NCR and other receptors responsible for NK cell activation such as NKG2D. Allogeneic CAR NK cells show good GvL killing and do not induce GvHD, but prolonged persistence is a challenge that must be overcome [39]. In fact, mouse studies showed that donor NK cell lysis of host antigen-presenting cells suppresses development of GvHD, as those are essential for donor T cell activation in GvHD induction [40]. Donor NK cells are also able to lyse syngeneic alloreactive GvHD-inducing T cells in vivo [41]. Currently, there are 26 trials investigating CAR NK cells for the treatment of hematological malignancies and solid tumors.

An interesting alternative that combines the best clinically relevant effector functions of both T cells and NK cells are CD3^+^CD56^+^ cytokine-induced killer (CIK) cells. CIK cells are ex vivo–activated cytotoxic T lymphocytes obtained from human peripheral blood or BM (autologous or allogeneic), or cord blood mononuclear cells, by sequential addition of IFN-γ, anti-CD3 antibody (OKT3) and high doses of recombinant human IL-2, reaching high numbers after 3 weeks in culture [42,43,44,45]. After these culture conditions, CIK cells express not only TCR/CD3 but also CD56, activating NK receptors; including NKG2D, DNAX accessory molecule-1 (DNAM-1) and low levels of NKp30, and inhibitory NK receptors; including inhibitory members of KIR and CD94-NKG2A [45,46,47]. Hence, cytotoxicity against a wide variety of tumor targets is performed through TCR/CD3, NKG2D, DNAM-1, NKp30 and the adhesion molecule lymphocyte function-associated antigen-1 [45]. CIK cell potential has been hypothesized to control both neoplastic relapses and viral infections in patients that received a transplant [45], and their infusion induces minimal GvHD in murine models and clinical trials [47,48,49]. CIK cells are pursued against a variety of tumors but one of their best applications is probably in the context of myeloid malignancies after allogeneic BM transplant [50]. CIK cells may be engineered to express cytokine genes improving proliferation rates and cytotoxic activities in clinical trials against a variety of tumors including lymphomas [51], and CAR providing additional killing capacity and versatility to their functional repertoire. The international registry on CIK cells (IRCC) was established in 2010 to collect and evaluate clinical trials using CIK cells for the treatment of patients of hematological malignancies and solid tumors, and reported a total of 106 clinical trials in 2020 [52].

## 2. Clinical Applications of MSC to Improve Outcomes in Hematopoietic Stem Cell Transplant

MSC are pursued to expand HSC ex vivo and improve engraftment after transplantation, for example of umbilical cord blood that has limited potential due to low cell doses. A pioneering study in 2012, studied engraftment in 31 adults with hematological malignancies who received transplants of two cord blood units, one of them containing cord blood that had been previously expanded ex vivo using cocultures with allogeneic STRO-3^+^ mesenchymal cells from healthy donors in a confluence of 70% or higher (NCT00498316). The cocultures had started fourteen days before transplantation, when the same fractions of the cord blood unit were seeded into 10 flasks containing the mesenchymal cells and serum-free medium with stem cell factor (SCF), FMS-like tyrosine kinase 3 ligand (FLT3L), thrombopoietin (TPO) and granulocyte colony stimulating factor (G-CSF). The results were compared with 80 historical controls who had received two units of unmanipulated cord blood. Coculture led to expansions of both total nucleated cells (12.2x) and particularly CD34^+^ cells (30.1x), so patients in this group received significantly higher cellular doses than those receiving unmanipulated cord blood. In patients with engraftment, cells engrafted faster i.e., 15 versus 24 days for neutrophils and 42 versus 49 days for platelets. There were also more patients with engraftment in the group receiving expanded cells i.e., 88% versus 53% for neutrophils on day 26 and 71% versus 31% for platelets on day 60 [53]. Following up these encouraging results, a disappointing pilot clinical trial was recently performed to assess feasibility and efficacy of expanding cord blood CD34^+^ cells for transplantation in patients of hematological malignancies, using a combination of SCF, TPO, FLT3L and insulin-like growth factor-binding protein 2 (IGFBP-2), and coculture with BM haplo-identical MSC obtained from the BM of a family member of the patient (NCT01624701). The coculture was performed on an 80–90% confluent BM MSC stromal layer (4th to 6th passage), and prior to addition of hematopoietic cells, BM MSC were checked for positive phenotypic expression of CD90, CD73 and CD105, and absence of CD45 and CD34. Two out of the three patients enrolled in the study died prematurely, which resulted in trial discontinuation. The authors reported suboptimal outcome regarding neutrophil engraftment, primarily due to complicated patient disease status at transplantation and low CD34^+^ cell infusion [54].

In an attempt to better sustain primitive HSC ex vivo, BM MSC were cultured as nonadherent mesenchymal spheres previous to cocultures [55,56]. Human mesenspheres were derived from CD45^−^CD31^−^Ter119^−^PDGFRα^+^CD51^+^ which characterizes a large fraction of BM nestin^+^ cells, CD45^−^CD31^−^CD71^−^CD146^+^CD105^+^nestin^+^ cells, or were simply grown from fetal or adult BM CD45^−^-enriched cells. Mesenspheres display a moderately undifferentiated phenotype, with low adherence to plastic and self-renewal capacity. They are enriched in HSC maintenance genes and promote expansion of cord blood CD34^+^ cells through secreted soluble factors. Human hematopoietic progenitors cocultured with mesenspheres are able to engraft in immunodeficient mice [55] and are serially transplantable, with increased long-term hematopoietic engraftment [56]. This technique has so far not been followed up with clinical trials.

Cotransplantation strategies of culture-expanded MSC and HSC from HLA-identical sibling donors after myeloablative therapy hypothesize that MSC could both facilitate engraftment and reduce GvHD. This strategy was feasible and seemed safe in patients of hematological malignancies, without MSC-associated toxicities [57]. In 2010, a phase I trial studied the side effects and best dose of donor MSC in treating 49 patients of hematological malignancies with acute or chronic GvHD after undergoing a donor stem cell transplant. No results were posted or published (NCT00361049). In 2019, random-effects model was applied to analyze 12 completed randomized controlled trials and 13 on-going trials including participants with a hematological condition who had undergone HSC transplant as treatment, and were randomized to MSC or no MSC to prevent or treat GvHD. This analysis included trials comparing different doses of MSC, MSC of different sources and MSC cotransplanted with HSC or administered post-HSC transplantation. The authors concluded that the overall quality of the studies is low, and the evidence from randomized controlled trials does not support MSC as an effective therapy for treating acute GvHD whereas they may reduce the risk of chronic GvHD [58].

## 3. Clinical Applications of Cellular Immunotherapy to Target the Malignant Hematopoietic Stem Cell

### 3.1. CAR T Cells

Immunotherapy, including adoptive cell therapy, is expected to improve prognosis of patients of hematological malignancies because it prevents use of the cytotoxic mechanisms of conventional chemotherapy [59]. It is highly versatile, and it can be delivered as induction or consolidation therapy, in combination or not with chemotherapy, HSC transplant, checkpoint inhibitors or other drugs targeting the tumor or aimed at modulating immune cell functions such as proliferation [37,60,61]. CAR T cells are successful in the treatment of relapsed/refractory (R/R) acute lymphoblastic leukemia (ALL) and B cell non-Hodgkin lymphoma [62,63,64]. However, 40–60% of patients relapse due to poor CAR T cell persistence or emergence of clones negative for CD19 [32]. Factors such as the single-chain spacer, the extracellular and costimulatory domains, and the CAR binding affinity influence CAR T cell function and persistence [32,65,66,67]. CD19 CAR (CAT) with lower affinity than the most frequent high-affinity binder used in clinical studies, FMC63, induced molecular remission in 12 out of 14 patients (NCT02443831) with R/R pediatric B cell ALL. CAR T cells showed enhanced expansion, and persistence was demonstrated in 11 of the 14 patients at last follow-up (up to 2 years) [32].

In AML, relapses are caused by treatment-selection of leukemic cells resistant to conventional chemotherapy, which are able to regenerate leukemia, i.e., leukemia stem cells (LSC) [68]. AML LSC may persist or diversify clonally after chemotherapy, driving progression to more aggressive forms of AML and fatal outcomes after relapse [69]. Thus, any potential curative therapy against a disease such as AML should ultimately aim at LSC eradication [68]. This has proven challenging though, as LSC are rare, heterogeneous among AML patients and share main features with healthy HSC, such as surface markers and functions such as dormancy and self-renewal [68,70,71]. CAR T cells engineered to target LSC have the potential to prevent relapse caused by LSC persistence, and selectivity against LSC will correlate directly with treatment safety. Nevertheless, results to date with AML CAR T clinical trials directed to target one single antigen such as CD33, CD123 and NKG2D ligands, reported limited therapeutic effects except for anti-CLL1 CAR T cells in 3 patients who reached complete remission after one month of treatment [62,72,73]. In preclinical models, promising data have been obtained through administration of activating anti-CD44 monoclonal antibody H90 into NOD/SCID mice transplanted with human AML cells. This treatment reduced leukemic engraftment, induced blast differentiation and eradicated functional LSC as evidenced by serial transplantations in vivo, due to reduced homing capacity of LSC into the BM microenvironment [74]. As a follow up, an exciting phase I/II clinical trial is currently recruiting R/R AML patients to monitor the safety and anti-leukemia therapeutic activity of autologous CD44v6 CAR T-cells (NCT04097301). In this trial, T cells are genetically engineered to express a CAR targeting the most abundant CD44 isoform expressed in AML patients, which predicts poor prognosis [75,76]. This is also a second generation of CAR T cells where the cells are further modified to express the suicide gene HSV-TK Mut2, activated in case of toxicity with ganciclovir.

AML heterogeneity may be a powerful reason for CAR T cell failure when directed against single antigens, due to incomplete targeting and clonal selection [77]. Combination of multiple antigens may be a good strategy to overcome this challenge [78]. In this direction, CAR T cells targeting antigens on LSC (CD123) and AML blasts (CD33) show relevant preclinical efficacy [78], and dual CAR T cells targeting CD123, CD33 or CLL1 are being tested in clinical trials (NCT03795779, NCT04156256 and NCT04010877) with promising results reported for CLL1-CD33 CAR T cells (NCT03795779) [79]. However, a clinical trial aimed at treating R/R AML patients using single CAR T or double CAR T cells with CD33, CD38, CD56, CD123, CD117, CD133, CD34 or Muc1 was recently suspended as the therapeutic effect was not as expected (NCT03473457). This indicates that further research is needed to tackle the most severe forms of the disease. Available CAR and their antigenic targets in the context of AML are summarized in Table 1.

### 3.2. NK and CAR NK Cells

In a recent phase I/II trial, 11 patients with R/R CD19^+^ non-Hodgkin lymphoma or chronic lymphocytic leukemia were administered with HLA-mismatched anti-CD19 CAR NK cells derived from cord blood (NCT03056339). NK cells were transduced with a retroviral vector expressing genes that encode anti-CD19 CAR, IL-15 and inducible caspase 9 as a safety measure. The cells were expanded ex vivo and administered in a single infusion after chemotherapy. Of the 11 patients, 7 reached complete remission and 1 partial remission, within 30 days. The infused CAR NK cells expanded and persisted at low levels for at least 12 months, without development of major toxic effects [80].

In AML, donor NK cells have a variety of interesting therapeutic properties including GvL killing and depletion of host T cells that promotes engraftment in the transplantation setting. Further, depletion of host T cells and antigen-presenting cells, together with other mechanisms such as production of IL-10, protect against GvHD [81,82]. Transplantation from NK alloreactive donors is a strong independent event that predicts survival in recipients of allogeneic HSC transplants [83,84]. NK cell transfer is a versatile strategy that reduces disease burden to make patients eligible for transplantation [85], consolidates engraftment after HLA-haploidentical HSC transplant [86,87] and reduces leukemia progression [88]. Adoptive NK cell transfer is a feasible consolidation therapy after chemotherapy, in the non-transplantation setting, able to induce durable remissions of AML [89,90,91]. As recently reviewed by Xu and Niu, more recent strategies aim at boosting the patient NK cell responses against AML by the use of monoclonal antibodies targeting tumor-associated proteins or NK cell inhibitory receptors, humanized bi-specific and tri-specific killer cell engagers, which are the recombinant single-chain variable fragments serving as immunological synapses between NK cells and tumor cells, cytokines or other immunomodulatory drugs [37].

CAR NK cells have been less explored in the context of AML, but CAR NK cells redirected against CD123 are able to bind and induce apoptosis of AML patient cells ex vivo [92]. Further, in the absence of a specific CAR target antigen, the innate and alloreactive potential of NK cells against AML provides for activity against target antigen negative clones. This together with fast availability and low toxicity makes CAR NK cell therapy a potentially suitable option for AML patients [93]. CD33 CAR NK-92 cells, cell line that can expand unlimited, were evaluated in a phase I clinical trial in 3 patients with R/R AML (NCT02944162). While multiple infusions of CD33 CAR NK-92 cells were safe, it is not possible to infer a therapeutic effect from this study [94]. The variety of NK cell sources, expansion methods, CAR designs and combination therapies together with the unique inherent functionality of NK cells, offer good versatility to potentially tackle the complexity of AML treatment in the future [93].

### 3.3. CIK and CAR CIK Cells

In clinical trials to treat AML, CIK cells have been infused in combination or not with allogeneic HSC transplantation [47]. Preliminary phase I/II clinical studies with cord blood-derived or autologous CIK cells showed little or none therapeutic value but confirmed low toxicity [95,96]. More recent data suggest that IL-15-activated CIK cells may sustain remission in a heterogeneous cohort of pediatric and adult leukemia patients after allogeneic HSC transplant [97]. Recently, CIK cells were engineered to express a CD33 CAR with the latest version of the non-viral Sleeping Beauty transposon system SB100X-pT4 [98]. SB-modified CD33 CAR CIK cells displayed antileukemic activity in vitro and in patient-derived xenograft models, when administered at both initial and advanced stages of the disease. CD33 CAR CIK cells were also effective against chemotherapy resistant/residual AML cells, further supporting future clinical development [98].

## 4. Considerations in Preclinical Studies of Disease Modelling and Treatment Efficacy Prediction

### 4.1. Healthy and Diseased HSC Reconstitution

**Figure 2:**
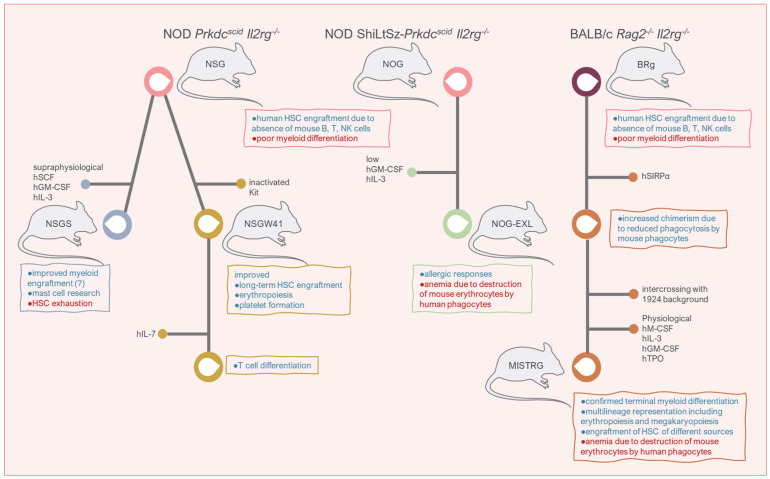
Immunodeficient mouse strains available for the study of HSC reconstitution. Background strains and additional genetic engineering are depicted. Advantages for research are shown in blue and disadvantages in red. The major limitation of all xenograft models is a mouse bone marrow microenvironment to support human HSC engraftment. h, human; SCF, stem cell factor; GM-CSF, granulocyte colony stimulating factor; IL, interleukin; SIRPα, signal-regulatory protein alpha; M-CSF, macrophage colony-stimulating factor; TPO, thrombopoietin; NK, natural killer.

Preclinical humanized models make it possible to study in vivo complex biological features of healthy and diseased HSC such as lineage commitment and self-renewal, and immune responses, without invasive procedures for the human donor. These models allow both disease modelling and prediction of efficiency of gene and cell therapy in a close to physiological setting. The mice used in these studies are immunodeficient and lack T, B and NK cells, to prevent graft rejection, usually in NOD or BALB/c backgrounds. Data obtained from these tools are highly valuable, but it should be considered in the context of their weaknesses to prevent misinterpretation. Selecting the best mouse model for the purpose of the research is a key step towards accuracy (Figure 2).

Strains in NOD (NOD *Prkdc^scid^ Il2rg*^−/−^; NSG) backgrounds support higher engraftment of human HSC compared to BALB/c-based recipients (BALB/c *Rag2*^−/−^ *Il2rg*^−/−^; BRg) [99]. NSG mice differ from NOG mice in the *Il2rg* mutation and the genetic background, but seem similar in biological features and xenograft outputs [100]. The NSG mouse was developed by backcrossing the *Il2rg*^−/−^ mouse resulting from a complete null mutation onto NOD/ShiLtSz-*Prkdc^scid^* mouse [101]. The NOG mouse was developed by backcrossing the *Il2rg*^−/−^ mouse resulting from a truncated intracellular signaling domain onto NOD/ShiLtSz-*Prkdc^scid^* mouse [102]. In NOG mice, the *Il2rg* mutant gene is expressed and produces a protein that binds cytokines but does not signal. Conversely, no *Il2rg* gene expression happens in NSG mice [16]. BRg mice expressing human signal-regulatory protein alpha (SIRPα) transgene show enhanced human chimerism relative to wild-type BRg, as result of inhibited phagocytosis of the human cells [103]. These models show low human myeloid cell differentiation due to evolutionary differences between mouse cytokines and growth factors and human receptors needed for myeloid differentiation [104].

The next generation of humanized mouse models, i.e., NSG-SGM3 (or NSGS), NSGW41, NOG-EXL and MISTRG, support human myelopoiesis at varying degrees and through different strategies. NSGS supply supraphysiological concentrations (2000–4000 pg/mL) of SCF, granulocyte-macrophage colony-stimulating factor (GM-CSF) and IL-3, expressed under the CMV promoter [104,105,106]. Some reports show that the transgenic overexpression of human cytokines induces human HSC exhaustion in these mice [104,107], whereas other authors demonstrate improved myeloid engraftment from normal human CD34^+^ progenitors and AML subtypes previously challenging to engraft [105,108]. Differences in technical protocols, and source and number of CD34^+^ cells transplanted may contribute to these discrepancies. In a recently improved protocol using ex vivo cultured CD34^+^ cells obtained from peripheral blood of patients with myelofibrosis (MF), xenotransplantation by X-ray guided intra-tibial injection into sublethally irradiated NSGS mice generated patient-derived xenografts (PDX) that reproduced the hallmarks of MF, including BM fibrosis. Genetic analysis showed maintenance of MF patient clonal architecture within the engrafted cell population and prediction of clonal progression to secondary AML for patients. The model was also useful for pharmacological target validation [109].

NSGW41 mice promote human engraftment by reducing mouse HSC through the W41/W41 inactivating mutation of the c-Kit gene, which opens the BM niche to human HSC engraftment and differentiation. This strategy achieves genetic preconditioning and prevents the use of irradiation, enabling robust, uniform, sustained and serially transplantable engraftment of human cord blood CD34^+^ progenitors [110]. NSGW41 mice also support improved human erythropoiesis and platelet formation from cord blood CD34^+^ progenitors compared to irradiated NSG recipients [111]. No remarkable limitations to this mouse model have been reported to date.

The NOG-EXL expresses human GM-CSF and IL-3 under the SV40 promoter on the NOG background, with expression levels in the range of 35 pg/mL for GM-CSF and 80 pg/mL for IL-3, and mimic human allergic responses [112]. MISTRG mice combine genetic preconditioning and cytokine-mediated support through knockin gene replacement. The base model is BRg on a mixed BALB/c and 12924 backgrounds, which also includes the transgene expressing human SIRPα to improve human HSC engraftment. In addition, MISTRG mice express physiological concentrations of human macrophage colony-stimulating factor (M-CSF), IL-3, GM-CSF and TPO, all under the endogenous mouse promoters except for GM-CSF that is under the human promoter control [113]. Of note, MISTRG PDX using BM-derived CD34^+^ cells from patients with myelodysplastic syndromes transplanted intrahepatically into sublethally irradiated newborn recipients, reproduced patient dysplastic morphology with multilineage representation, including erythro- and megakaryopoiesis. MISTRG MDS-PDX replicated the original patient sample genetic complexity, could be propagated via serial transplantation and was useful for pharmacological target validation [114].

Mouse red blood cells are susceptible of destruction by human phagocytes, resulting in anemia in both NOG-EXL and MISTRG mice [113,115]. In the original description of MISTRG mice, anemia was lethal when engraftment reached ∼50% [113]. In a recent comparative study using NSGS, NSGW41 and MISTRG mice transplanted with fetal CD34^+^ cells, anemia was confirmed in MISTRG recipients with negligible effects on red blood cell counts on the other two strains. However, there was no significant difference in survival among groups. Discrepancy with MISTRG previous report was explained on the basis of the lower dose of radiation use (80 cGy versus 150 cGy), lower numbers of fetal CD34^+^ cells transplanted (20,000–50,000 versus 100,000 cells) and rederivation of the line which potentially eliminated a pathobiont that induced anemia-related lethality [107]. In accordance with some previous studies, the authors further concluded that NSGS mice may be used for mast cell research [116], but they are defective in HSC maintenance and myeloid cell maturation. They also confirmed NSGW41 mice are suitable models for human HSC studies, and proposed MISTRG mice as a supportive model for engraftment and maintenance of HSC of a variety of sources, i.e., fetal, newborn and adult, and differentiation to terminal monocytes and macrophages [107].

Additional comparative studies are highly recommended, particularly in the contexts of gene editing and cell therapy. Despite its limitations, NSG is the most common strain used in these contexts to date. For example, NSG mice have been used to test the efficiency of gene editing in human long-term HSC using a barcoding strategy to track the edited clones. This elegant strategy showed that editing activates p53, which reduces the HSC clonal repertoire in vivo, but does not affect multilineage reconstitution and self-renewing properties [117]. Transient p53 inhibition restored polyclonal graft composition and combined E4orf6/7 expression and p53 inhibition increased homology-directed repair editing efficiencies to up to 50% in long-term HSC, with no functional perturbations [117].

Importantly, the greatest limitation of xenograft models is a mouse BM microenvironment to support human HSC engraftment and sustain human hematopoiesis. As of today, there is insufficient knowledge of the murine HSC niche cell types and molecular interactions involved [118]. For example, mouse CXCL12 binds and signals through human CXCR4, whereas other soluble factors such as osteopontin expressed by stromal cells including MSC and its receptors expressed on HSC, show only moderate protein identity between mouse and human, suggesting little contribution to human HSC maintenance in the mouse HSC niche [118,119,120]. A potential strategy to overcome this milestone is to humanize the HSC niche. After intra-tibia transplantation in NOD/SCID mice, human MSC engraft in BM and integrate into the microenvironment, differentiating into pericytes, myofibroblasts, BM stromal cells, osteocytes in bone, bone-lining osteoblasts and endothelial cells [121]. The presence of human MSC resulted in increased human hematopoietic output and numbers of CD34^+^ cells in mouse BM after transplantation of human cord blood CD34^+^ cells [121]. Later, human induced MSC transduced with platelet derived growth factor subunit B (*PDGFB*) by lentiviral transduction cotransplanted intra-tibia in NOD/SCID or NOG mice with human cord blood CD34^+^ cells promoted superior engraftment and proliferation of human hematopoietic cells, and supported self-renewal as demonstrated by remarkable engraftment into secondary recipients, versus no MSC or GFP-MSC [25]. Human PDGFB-MSC showed better survival and proliferation than GFP-MSC in the mouse BM [25].

### 4.2. GvHD

Chronic GvHD induced by human T cells derived from CD34^+^ cells in humanized mice has been explored scarcely. It was initially characterized as a systemic autoimmune disease ≥24 weeks post patient CD34^+^ cell receipt, which involved lung, liver, skin, gingiva and intestine in NSG mice. Affected mice received cells with HLA haplotypes that induce susceptibility for psoriasis in humans [122]. In these experiments, engraftment was supported by the weekly administration of a fusion protein providing IL-7 for stimulating T cell development in a stable form with bioavailability over 7 days after injection [122]. NSG mice engrafted with CD34^+^ cells from cord blood with chronic GvHD high-risk HLA haplotypes (C*0602 and C*0401) developed chronic GvHD 18 to 39 weeks post-engraftment, with symptoms that included severe weight loss, impaired movement, severe ruffling, facial/full body alopecia and scaly skin. This was accompanied by remarkable human CD45^+^ engraftment, particularly T cells [123].

NSGW41 mice were recently optimized for in vivo human T cell differentiation by expression of human IL-7 under the control of mouse IL-7 regulatory elements, which then supports a better balanced human immune system output without evidenced pathological events [124]. Future work will elucidate the susceptibility of these mice to chronic GvHD, and their utility for the study of this condition.

### 4.3. Immunotherapy

Humanized PDX models are used to predict the efficiency of immunotherapy. However, the function and antitumor activity of CAR T cells have been characterized mainly in vitro, given that PDX models provide a host environment that differs from patients due to their immunodeficient nature and presence of allogeneic/xenogeneic immune responses [125]. Basic NSG mice can be engrafted with different types of human leukocytes and CD34^+^ cells, obtained from BM, umbilical cord blood, fetal liver or thymus. Successful engraftment is at 25% or more human CD45^+^ cell chimerism in peripheral blood of recipient mice. Specific PDXs from solid tumors can then be inserted into the humanized mice, and an immunotherapeutic agent can be administered for testing [126].

In leukemia immunotherapy, another limitation that may result in discrepancies in treatment efficacy between patients and humanized PDX models is the use of cell lines as opposed to primary leukemia cells [127,128,129]. In many cases though, studies combine generation of CAR T cells derived from patients, such as B cell ALL patients, to test antitumor responses against cell lines in NSG models. This strategy has led to interesting observations such as the positive correlation between the proportion of stem cell memory T cells in the final CAR T cell product with the in vivo expansion of CAR T cells and long-term remission [128]. To mimic faithfully patient response to immunotherapy, the ideal PDX model should model patient immune responses in presence of autologous leukemia. Some elegant efforts have been taken in the direction. For example, for humanized mouse preparation, NSG mice were conditioned with sublethal irradiation, and transplanted intravenously with human CD34^+^ fetal liver cells and under kidney capsule with fetal thymus from the same donor. Human T cells were isolated from humanized mouse spleens, and then transduced with anti-CD19 CAR- or control CAR-expressing lentiviral or retroviral particles, and used immediately after expansion for adoptive immunotherapy in leukemic humanized mice. The same human fetal liver-derived CD34^+^ cells, previously transduced with retroviruses expressing MLL-AF9, were transplanted into different NSG mice to develop human B cell ALL. Leukemic humanized mice were generated using transplants of these human B ALL cells into the humanized mice. The result is a valuable in vivo system to model antitumor responses of CAR T cells, with potential to help identify new more efficient CARs [125]. Future improvements in xenograft settings are needed, particularly in the context of leukemia and immunotherapy.

## 5. Conclusions

HSC transplant has considerably improved survival in patients of previously deadly diseases such as AML. Currently, HSC engraftment requires preconditioning through heavy irradiation or chemotherapy, which makes this therapy not suitable for the older patient, and has damaging consequences for the HSC niche in the BM and long-term HSC reconstitution. The research agenda should include exploring alternative, less toxic, ways of preconditioning and improved understanding of the BM HSC niche. To date, allogeneic HSC transplantation is the most successful setting to prevent relapse, but it increases the likelihood of GvHD, a condition that can be life threatening. In this context, MSC are pursued mainly to promote HSC niche reconstitution, HSC engraftment and expansion, and GvHD suppression. Results are somewhat disappointing, mainly due to our poor understanding of MSC immunophenotype and their fine-tuned roles in tissue remodeling and paracrine regulatory interactions as well as absence of standardization of their culture conditions to preserve their function. Future improved detection methods and characterization of their nature will allow to determine their true potential as cell therapy in these contexts and expand their application to promote immunotherapy, or HSC niche reconstitution in additional scenarios such as myelodysplastic syndromes and other BM failure syndromes.

Cellular immunotherapy holds the promise of improved cure rates, providing specificity and less toxicity as compared to chemotherapy. Neurotoxicity and GvHD are still challenges for the broad application of CAR T cells, which are currently tackled from extensive characterization of these powerful cells and their responses to the engineered expression of suicide genes. The next generation of CAR T cells will be further engineered to express receptors that will allow their regulation from the BM microenvironment. To harness the full potential of this strategy in the context of AML will require better understanding of the hijacking of the BM HSC niche exerted by LSC. Further, CAR T cells directed against single antigens are unsuccessful in AML likely due to heterogeneity and insufficient knowledge of LSC, which are subject of current extended research. Dual blast and LSC targeting is being pursued in several clinical trials at the moment with some promising results already reported. CAR NK cells are a more recent approach with advantages over CAR T cells, including no induction of GvHD, dual killing capacity by CAR-dependent and innate pathways as well as fast availability. Their persistence in vivo seems to be a challenge that may be overcome by improved understanding of the regulatory mechanisms of these cells through the microenvironment, and this should be subject of future investigation. As of today, there are more than 800 clinical trials using CAR T cells in hematology and oncology, and 26 investigating CAR NK cells in hematological malignancies and solid tumors. Other forms of immunotherapy are also available such as NK cells, CIK cells and CAR CIK cells and show promising results in the context of AML. Cellular immunotherapy can be strengthened or modulated by further gene engineering, and combination or not with chemotherapy, HSC transplant, checkpoint inhibitors or other drugs targeting the tumor or aimed at modulating immune cell functions. It is a powerful strategy that offers the versatility required to tackle complex diseases. We are witnessing the birth of the hematology of tomorrow.

Finally, disease modelling and treatment efficacy predictions in hematology rely on the use of preclinical models. Data derived from these models are highly valuable, but it should be considered in the context of their limitations to prevent misinterpretation. Careful choice of the preclinical model among those available, according to the purpose of the study, is highly recommended. Genetic preconditioning in NSGW41 is a promising strategy that allows the study of cell behavior in a native microenvironment, not subjected to previous irradiation. However, the challenge of a mouse BM microenvironment to support and regulate human stem and immune cells is a significant milestone that still must be overcome. There is insufficient understanding of the mouse HSC niche, and its cellular and molecular alterations in the context of immunodeficient mice as well as differences among models. The field will benefit substantially from further research on improved preclinical models including humanized microenvironments in mice.

## Figures and Tables

**Table 1:** Available CAR in AML and examples of clinical trials.

Antigenic Target	Cellular Immunotherapy	Clinical Trial
Single strategies		
CD33	CAR T	NCT04010877, NCT03971799
	CAR NK-92	NCT02944162
CD123	CAR T	NCT03796390, NCT03585517, NCT04014881, NCT03556982, NCT03766126, NCT04010877
NKG2 ligands	CAR T	NCT04167696
CLL1	CAR T	NCT04010877, NCT04219163
CD44v6	CAR T	NCT04097301
CD7	CAR NK-92	NCT02742727
Dual strategies		
CD123/CD33	CAR T	NCT04156256
CLL1/CD33	CAR T	NCT03795779, NCT04010877
CLL1/CD123	CAR T	NCT03631576, NCT04010877
Single/dual strategies		
Muc1/CD33/CD38/CD56/CD123/CD117/CD133/CD34	CAR T	NCT03473457
Multi strategies		
Muc1/CLL1/CD33/CD38/CD56/CD123	CAR T	NCT03222674
